# Influence of Autocorrelated Rhythmic Auditory Stimulations on Parkinson’s Disease Gait Variability: Comparison With Other Auditory Rhythm Variabilities and Perspectives

**DOI:** 10.3389/fphys.2020.601721

**Published:** 2020-12-23

**Authors:** Alexis Lheureux, Thibault Warlop, Charline Cambier, Baptiste Chemin, Gaëtan Stoquart, Christine Detrembleur, Thierry Lejeune

**Affiliations:** ^1^Institute of NeuroScience, Université catholique de Louvain, Woluwe-Saint-Lambert, Belgium; ^2^Department of Physical and Rehabilitation Medicine, Cliniques universitaires Saint-Luc, Woluwe-Saint-Lambert, Belgium; ^3^NeuroMusculoSkeletal Lab (NSMK), Institut de Recherche Expérimentale et Clinique, Université catholique de Louvain, Woluwe-Saint-Lambert, Belgium

**Keywords:** gait disorders, rhythmic auditory stimulations, cueing, gait variability, long range autocorrelations, Parkinson’s disease, fractals

## Abstract

Parkinson’s Disease patients suffer from gait impairments such as reduced gait speed, shortened step length, and deterioration of the temporal organization of stride duration variability (i.e., breakdown in Long-Range Autocorrelations). The aim of this study was to compare the effects on Parkinson’s Disease patients’ gait of three Rhythmic Auditory Stimulations (RAS), each structured with a different rhythm variability (isochronous, random, and autocorrelated). Nine Parkinson’s Disease patients performed four walking conditions of 10–15 min each: Control Condition (CC), Isochronous RAS (IRAS), Random RAS (RRAS), and Autocorrelated RAS (ARAS). Accelerometers were used to assess gait speed, cadence, step length, temporal organization (i.e., Long-Range Autocorrelations computation), and magnitude (i.e., coefficient of variation) of stride duration variability on 512 gait cycles. Long-Range Autocorrelations were assessed using the evenly spaced averaged Detrended Fluctuation Analysis (α-DFA exponent). Spatiotemporal gait parameters and coefficient of variation were not modified by the RAS. Long-Range Autocorrelations were present in all patients during CC and ARAS although all RAS conditions altered them. The α-DFA exponents were significantly lower during IRAS and RRAS than during CC, exhibiting anti-correlations during IRAS in seven patients. α-DFA during ARAS was the closest to the α-DFA during CC and within normative data of healthy subjects. In conclusion, Isochronous RAS modify patients’ Long-Range Autocorrelations and the use of Autocorrelated RAS allows to maintain an acceptable level of Long-Range Autocorrelations for Parkinson’s Disease patients’ gait.

## Introduction

Several physiological signals, apparently randomly organized, are in fact governed by dynamic phenomena organized between order and disorder (Goldberger et al., 2002; Hu et al., 2004). This complex self-organization is the result of multiple interactions between different elements of the system (Delignieres and Marmelat, 2012; Stergiou, 2016). Such complexity of organization is visible in the study of the temporal organization of human gait (Hausdorff et al., 2001; Ivanov et al., 2009; Stergiou and Decker, 2011). Indeed, gait variability organization is the result of multiple interactions between internal (nervous system, biomechanical structure) and external (proprioceptive, visual, auditory information) components (Hausdorff et al., 2000, 2001; Ashkenazy et al., 2002; Gates et al., 2007; Dotov et al., 2017; Lheureux et al., 2020). The study of the magnitude (using linear mathematical methods) and of the temporal organization (using nonlinear mathematical methods) constitute complementary ways to assess gait variability, and stride duration variability in particular (Delignieres et al., 2006; Stergiou and Decker, 2011). Stride duration varies in the short and long term according to a complex dynamic of temporal variations (Hausdorff, 2007). These variations present Long-Range Autocorrelations (LRA) (Stergiou et al., 2006; Hausdorff, 2007; Cavanaugh et al., 2017) involving a long-memory process which means that every stride duration depends on the duration of the previous strides (Hausdorff et al., 2001; Hausdorff, 2007). Nevertheless, the origin and control of LRA in human locomotion remain elusive. While some attribute their origin to biomechanical structures (Gates et al., 2007), the most common theory is that LRA reflect a control from the central nervous system (Hausdorff et al., 2000). Indeed, a degradation of LRA has been shown in Parkinson’s and Huntington’s disease suggesting that the phenomenon would come from supraspinal centers (Hausdorff et al., 1997; Hausdorff, 2009; Warlop et al., 2016). Other theories suggest the existence of Central Pattern Generators (CPGs) at spine level to describe dynamics of human gait (Ashkenazy et al., 2002). After years of research, some authors have suggested that LRA are markers of healthy stable but still adaptive gait and a breakdown of LRA would be a sign of gait disorders and loss of adaptability as suggested in Parkinson’s Disease (PD) (Goldberger et al., 2002; Stergiou and Decker, 2011; Cavanaugh et al., 2017).

Among motor symptoms, PD patients suffer from gait disorders such as shorter step length, reduced gait speed, and increased randomness in temporal organization of gait (Hausdorff, 2007). Indeed, a breakdown of LRA (reduced fractal scaling α exponent) in stride duration variability in PD gait and strong correlations between LRA, disease severity and postural instability were demonstrated (Ota et al., 2014; Warlop et al., 2016). Diminished α exponent would be linked to basal ganglia degeneration (Hausdorff et al., 1997; Goldberger et al., 2002; Hausdorff, 2007; Sarbaz et al., 2012) involved in the regulation of gait, posture and rhythm control (Hausdorff, 2009; Takakusaki, 2017). LRA measurement would therefore be a biomarker of gait instability and risk of falling, which is of particular clinical interest in PD (Hausdorff, 2009; Warlop et al., 2016). Given that PD patients’ gait disorders are partially responsive to medication (Nieuwboer et al., 2007; van der Kolk and King, 2013), there is a need for rehabilitative approach (Tomlinson et al., 2014).

As previously mentioned, gait is organized according to the interactions between internal and external components, such as proprioceptive, visual or auditory information. Rhythmic Auditory Stimulations (RAS) acting as an external cue by means of a metronome, have been studied for years for their effects on PD patients’ gait (Ghai et al., 2018). This cueing would act like an external rhythm generator bypassing the basal ganglia that can no longer properly act as an internal rhythm generator in PD patients (Nieuwboer et al., 2007). It is then suggested that a broader use of isochronous RAS should be beneficial in PD patients’ gait rehabilitation (Spaulding et al., 2013). However, it has been demonstrated that the use of isochronous RAS modify LRA in young (Kaipust et al., 2013; Marmelat et al., 2014) and older (Kaipust et al., 2013) healthy subjects and in PD patients (Hove et al., 2012; Dotov et al., 2017).

Some authors tried to study the effects of autocorrelated RAS (i.e., rhythm variability presenting with LRA) on healthy subjects’ (Kaipust et al., 2013; Marmelat et al., 2014) and PD patients’ LRA computed from gait tasks (Dotov et al., 2017; Marmelat et al., 2020). Although some studies showed that autocorrelated RAS are beneficial for stride duration variability, these results should be interpreted with caution given the short acquisition times used (except for Marmelat et al., 2020). Indeed, a long acquisition time (at least 512 gait cycles) is necessary to show the presence of LRA with a high level of evidence (Crevecoeur et al., 2010; Warlop et al., 2017; Marmelat et al., 2018; Marmelat and Meidinger, 2019; Ravi et al., 2020). Series length has a clear effect on the statistical precision and the sensitivity of scaling exponents (Warlop et al., 2017). Shorter series lengths lead to loss of accuracy and are too short to be statistically different from short-range correlated processes (Warlop et al., 2017; Marmelat and Meidinger, 2019). In this sense, there is a risk that LRA computations using short series could not reflect the results on long series (Warlop et al., 2017; Marmelat and Meidinger, 2019).

Dotov et al. (2017) and Marmelat et al. (2020) tested autocorrelated RAS on PD patients. Dotov et al. (2017) showed that autocorrelated RAS allow to maintain similar level of LRA (similar α exponent) than during their control condition without RAS and that isochronous RAS deeply modify LRA. However, their findings require confirmation given their short acquisition time (5 min per condition). It should also be noted that their method to produce their autocorrelated RAS remains unknown and that their RAS frequency was set 10% faster than each participant’s preferred cadence, which will both differ in this present study. Also, Dotov et al. (2017) used a 21.6 m track, probably imposing a constant strong steering while our 42 m track should allow smoother steering. This could be of importance since steering is known to influence LRA (Dotov et al., 2016). Unlike Dotov et al. (2017), Marmelat et al. (2020) found significantly higher α exponent values during their autocorrelated RAS condition (the 1:1 step-to-beat ratio version) compared to their control condition. In their study, music was used to deliver RAS when a simple beat will be used in this study. This is not negligible since music is composed of multiple “layers” including the rhythmic beat itself, melody and harmony. Furthermore, Marmelat et al. (2020) used an α exponent = 1.02 as a reference while an α exponent similar to normative data of healthy subjects will be used in this study.

The purpose of this pilot study is to analyze the effects of three different RAS (isochronous, random and autocorrelated RAS) on PD patients’ spatiotemporal gait parameters and stride duration variability (magnitude and temporal organization) using suitable acquisition times to compute LRA. Our main hypothesis is that the autocorrelated RAS will be more efficient than the isochronous RAS and the random RAS to maintain LRA in the temporal organization of stride duration variability of PD patients.

## Methods

### Ethics, Consent, and Permissions

This study obtained ethical approval from the local ethical board (B403201318916/clinicaltrials.gov registration: NCT03716674). Participants gave written informed consent prior to data collection and this study adhered to the Declaration of Helsinki.

### Participants

Nine PD patients were included in this pilot study. Inclusion criteria were: PD diagnosis made according to United Kingdom Brain Bank criteria (Hughes et al., 1992), stages I–III on the modified Hoehn and Yahr scale (Goetz et al., 2004), ability to walk for a minimum of 512 gait cycles (±15 min) in a row without walking aids, stable dopaminergic medication for a minimum of 4 weeks before assessments, no other pathology that could interact with motor capacities and gait performance, a minimum of 24/30 on the Mini-Mental State Examination (MMSE) (Dick et al., 1984).

Patients’ anthropometric and clinical characteristics are summarized in Table 1.

**TABLE 1:** Characteristics of the study population.

	PD patients (*n* = 9)
Age (years)	66.6 (±8.17)
Height (cm)	170.1 (±11.96)
Weight (kg)	69.7 (±19.43)
Gender (male/female)	4/5
Hoehn and Yahr (modified)	2 [1–2.5]
Mini Mental State Examination (/30)	28.9 (±0.92)
10 Meter Walk Test (m.s^–1^)	1.3 (±0.22)
ABC Scale (%)	77.3 (±13.94)
BESTest (%)	78.7 (±10.62)
MDS-UPDRS (/260)	45.7 (±25.72)
PIGD (/20)	3.7 (±2.69)
Number of falls (#/6 months)	0.6 (±1.33)

**Mean (±SD), Median (interquartile range); ABC-Scale, Activities-Specific Balance Confidence Scale; BESTest, Balance Evaluation Systems Test; MDS-UPDRS, Movement Disorder Society sponsored Unified Parkinson’s Disease Rating Scale Revision; PIGD, Postural Instability and Gait Disorder.**

### Stimulus

In addition to a control walking condition without cueing [i.e., Control Condition (CC)], three conditions involved walking with three different RAS: Isochronous RAS (IRAS), Random RAS (RRAS), and Autocorrelated RAS (ARAS). Each of them was composed with an internally developed software (Matlab 2014R, Mathworks, United States) and adapted to each patient according to their spontaneous cadence determined before the experiment with 10 Meters Walking Tests. For each patient, these three RAS had the same mean interbeat duration [Mean (s) = 0.54 ± 0.05] but different magnitude (i.e., coefficient of variation, CV) and temporal organization of rhythm variability. During IRAS, the RAS presented no variation of the interbeat intervals [CV (%) = 0.00 ± 0.00]. During ARAS, autocorrelated interbeat intervals were used with α exponent similar to healthy subjects’ data of α exponent measured during gait in a previous study using the evenly spaced averaged Detrended Fluctuation Analysis [α-DFA = 0.78 ± 0.00; CV (%) = 0.92 ± 0.00] (Warlop et al., 2018). During RRAS, a random variability of the interbeat intervals was used and obtained by shuffling the interbeat intervals used for each patient during the ARAS [α-DFA = 0.46 ± 0.00; CV (%) = 0.92 ± 0.00].

### Procedure

Prior to data collection, patients listened to the RAS and were asked to mark the rhythm with a finger tapping task to ensure that the rhythm of the RAS was detected. Then, each patient walked in the four conditions in a randomized order. During RAS conditions, patients were listening to the RAS through earphones by the mean of a MP3 player. Standardized instructions to “walk accordingly to the proposed rhythm,” the foot contact of each step corresponding to each beat of the metronome, were given to each participant. During each condition, patients walked around on an oval indoor track of 42 m during ±15 min. The heading direction (clockwise or counterclockwise) was randomized but each patient kept the same heading direction for all conditions. A maximum of two conditions were tested during 1 day with a minimum break of 5 min between each of the conditions to avoid a fatigue effect and to limit a potential order effect. Patients came back a second day (2–14 days apart from the first session) to perform the other two conditions. The experiment was always performed at the same time of the day for the same patient during ON phase of dopaminergic treatment to avoid drug effect.

### Data Acquisition

Two unidimensional accelerometers were taped on patients’ both lateral malleoli in the antero-posterior direction and connected to a recording device (Vitaport 3 – Temec Instruments B.V., Kerkrade, The Netherlands) attached to the patients’ waist. Ankle accelerations were recorded while walking at a sample of 512 Hz and were then transferred onto a computer. Each peak of acceleration, corresponding to each foot contact, was detected by an homemade software to determine stride durations (i.e., peak detection method; Terrier and Dériaz, 2011).

### Functional Assessment

Functional assessment was performed before the beginning of the first walking condition. A 10 Meters Walk Test was used to calculate the patients’ spontaneous cadence used to individually adapt the RAS. Patients also completed the Activities-Specific Balance Confidence Scale (ABC scale) (Powell and Myers, 1995) to assess their balance-confidence, the Balance Evaluation Systems Test (BESTest) (Maia et al., 2013) to test their balance, the Movement Disorder Society sponsored Unified Parkinson’s Disease Rating Scale Revision (MDS-UPDRS) (Goetz et al., 2008) to globally assess the severity of motor and non-motor symptoms, the Postural Instability and Gait Disorder (PIGD) (Parashos et al., 2015) which groups five items (#13–15 and #29–30) of the UPDRS and the number of falls during the last 6 months before the experiment (see Table 2).

**TABLE 2:** Absolute mean values of the spatiotemporal gait parameters and stride duration variability assessed during Control Condition (CC), Isochronous Rhythmic Auditory Stimulations condition (IRAS), Random Rhythmic Auditory Stimulations condition (RRAS), and Autocorrelated Rhythmic Auditory Stimulations condition (ARAS) and comparison between these walking conditions.

	CC	IRAS	RRAS	ARAS
Gait speed (m.s^–1^)	1.30 (±0.26)	1.29 (±0.25)	1.27 (±0.24)	1.27 (±0.26)
Step length (m)	0.69 (±0.10)	0.69 (±0.10)	0.68 (±0.10)	0.68 (±0.11)
Cadence (#steps.min^–1^)	113.01 (±8.01)	112.03 (±9.22)	111.89 (±9.06)	111.86 (±9.07)
Mean stride duration (s)	1.07 (±0.07)	1.08 (±0.09)	1.08 (±0.09)	1.08 (±0.09)
Coefficient of variation (%)	1.87 (±0.71)	1.53 (±0.34)	1.66 (±0.25)	1.78 (±0.40)
α-DFA	0.76 (±0.09)^†^	0.44 (±0.09)*^†^	0.54 (±0.18)*^†^	0.66 (±0.09)*
Z-score α-DFA	−0.33 (±0.63)	−2.64 (±0.66)	−1.91 (±1.30)	−1.05 (±0.64)

*Absolute value is expressed as mean (±SD).*

α-DFA, α exponent calculated using the evenly spaced averaged version of the Detrended Fluctuation Analysis.

*Significant difference with CC (p <*0.05).*

*^†^Significant difference with ARAS (p < 0.05).*

### Gait Assessment

Gait was assessed through the measurement of the spatiotemporal gait variables, the magnitude and the temporal organization of the stride duration variability.

The data was extracted from 512 consecutive gait cycles which is recommended to assess temporal organization of the stride duration variability (Crevecoeur et al., 2010; Warlop et al., 2017; Ravi et al., 2020).

Spatiotemporal gait parameters were assessed as follow:

$$Gaitspeed\left(m.s^{-1}\right)=\frac{T\,o\,t\,a\,l\,w\,a\,l\,k\,i\,n\,g\,d\,i\,s\,t\,a\,n\,c\,e\,\left(m\right)}{T\,o\,t\,a\,l\,a\,c\,q\,u\,i\,s\,i\,t\,i\,o\,n\,t\,i\,m\,e\,\left(s\right)};$$

$$Cadence\left(\#steps.min^{-1}\right)=\frac{T\,o\,t\,a\,l\,n\,u\,m\,b\,e\,r\,o\,f\,s\,t\,e\,p\,s\,\left(\#\right)}{T\,o\,t\,a\,l\,a\,c\,q\,u\,i\,s\,i\,t\,i\,o\,n\,t\,i\,m\,e\,\left(m\,i\,n\right)};$$

$$S\,t\,e\,p\,l\,e\,n\,g\,t\,h\,\left(m\right)=\frac{Gaitspeed\left(m.{\text{s}}^{-1}\right)}{C\,a\,d\,e\,n\,c\,e\,\left(H\,z\right)};$$

$$M\,e\,a\,n\,s\,t\,r\,i\,d\,e\,d\,u\,r\,a\,t\,i\,o\,n\,\left(s\right)=\frac{T\,o\,t\,a\,l\,a\,c\,q\,u\,i\,s\,i\,t\,i\,o\,n\,t\,i\,m\,e\,\left(s\right)}{T\,o\,t\,a\,l\,n\,u\,m\,b\,e\,r\,o\,f\,s\,t\,r\,i\,d\,e\,s\,\left(\#\right)}.$$

To assess magnitude of stride duration variability, CV was calculated using the mean stride duration and standard deviation (SD) : $CV\left(\%\right)=\left[\frac{S\,D}{m\,e\,a\,n}\right].100$.

Temporal organization of stride duration variability was assessed by LRA computation using the evenly spaced averaged DFA (Almurad and Delignières, 2016) to obtain α-DFA exponent. This method was chosen among others given its robustness regarding stationary and non-stationary processes (Phinyomark et al., 2020; Ravi et al., 2020).

The original time series size was N ≥ 512. Then, the series was divided in subsets of size t, from *t* = 10 to *t* = N/2. The number of points used to calculate the slope in evenly spaced averaged DFA was based on Almurad and Delignières (2016). This method consists in selecting the data used for the regressions as follows:

$$\{\begin{array}{cc} n_{1}= & n_{min}\\ n_{i}= & [n_{i-1} \end{array}\begin{array}{c} 10\frac{log_{10}(n_{max})-log_{10}(n_{min})}{k-1}] \end{array}$$

Where k represents the number of points to include in the diffusion plot, the k interval lengths are noted [n_*i*_ (*i* = 1, 2,…k)] and n_*max*_ and n_*min*_ correspond to the maximum and the minimum interval lengths. We used n_*min*_ = 10, n_*max*_ = N/2 and *k* = 18 to follow the study by Almurad and Delignières (2016). After selecting evenly spaced data points, the linear regressions can be performed on these selected points (evenly spaced), or on the average data across the data points that are between selected points (evenly spaced averaged).

LRA are present when α-DFA is between 0.5 and 1 which implies persistence in the variations meaning that large stride duration fluctuations tend to be followed by other large fluctuations, and vice-versa. α-DFA <0.5 is the sign of anti-persistence and α-DFA = 0.5 corresponds to randomness (i.e., white noise). An α-DFA = 1 (i.e., 1/f noise) is the boundary between stationarity and non-stationarity (Hausdorff et al., 1996). In this context, 1/f noise is interpreted as “a ‘compromise’ between the complete unpredictability of white noise (α = 0.5) (very rough ‘landscape’) and the very smooth ‘landscape’ of Brownian noise (α = 1.5)” (Peng et al., 1995). Then, α-DFA = 1 is considered as the optimal state of variability characterizing healthy gait according to the theoretical framework of optimal movement variability (Harrison and Stergiou, 2015; Ravi et al., 2020).

### Statistical Analysis

A power analysis was made based on a previous study of Dotov et al. (2017) using PASS software, in the idea of performing a one-way repeated measures ANOVA. Total sample of nine participants achieved 80% power to detect differences among the means vs. the alternative of equal means using an *F*-test with a 0.05 significance level.

Statistical analyses were conducted using Sigmaplot 13.0. After verification with a Shapiro-Wilk normality test, a one-way repeated measures ANOVA was applied to determine the effect of each RAS type on all the gait parameters. When a significant difference between groups was detected, a *post-hoc* Tukey Test was performed. Effect size between conditions regarding α exponents was assessed using Cohen’s d. For the linear measures of stride duration variability, the results of the coefficient of variation (CV) did not pass the normality test (*p* < 0.05). A Friedman Repeated Measures ANOVA on ranks was then applied. The results were considered statistically different for *p* < 0.05. Results of α-DFA were also normalized using Z-scores. The mean α-DFA of the healthy population used to compute Z-scores was taken from the meta-analysis of Ravi et al. (2020) (α-DFA = 0.81 ± 0.14). They studied the effect of PD on α-DFA by compiling the results of 7 studies including a total of 177 PD patients and 135 healthy subjects.

## Results

### Spatiotemporal Gait Variables

No significant difference was found between each condition for gait speed [*F*(3, 8): 1.427; *p* = 0.260], gait cadence [*F*(3, 8): 0.709; *p* = 0.556], step length [*F*(3, 8): 1.224; *p* = 0.323], and mean stride duration [*F*(3, 8): 0.674; *p* = 0.577] (Table 2).

### Stride Duration Variability

Regarding the magnitude of the stride duration variability, CV [*F*(3, 8): 1.787; *p* = 0.177] was similar in all four conditions (Table 2).

**FIGURE 1:**
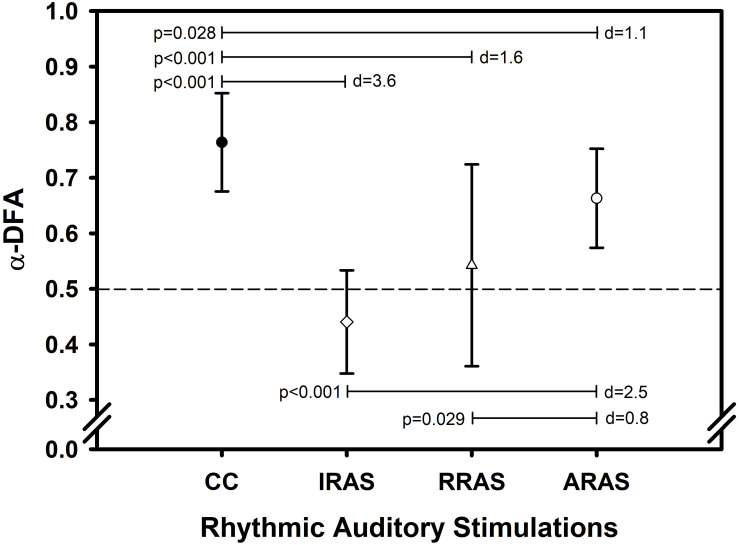
Error bars comparing mean values ± standard deviation of α-DFA obtained during each condition. *X*-axis represents the walking conditions. *Y*-axis represents mean α exponent value during each condition and calculated using the evenly spaced averaged version of the Detrended Fluctuation Analysis (α-DFA). Horizontal lines represent significant differences between conditions. Each *p*-value is indicated on the left of each line and Cohen’s d represents the effect size between conditions on the right (d). The black dashed line placed at 0.5 on the *Y*-axis delimit the area above which there are LRA.

**FIGURE 2:**
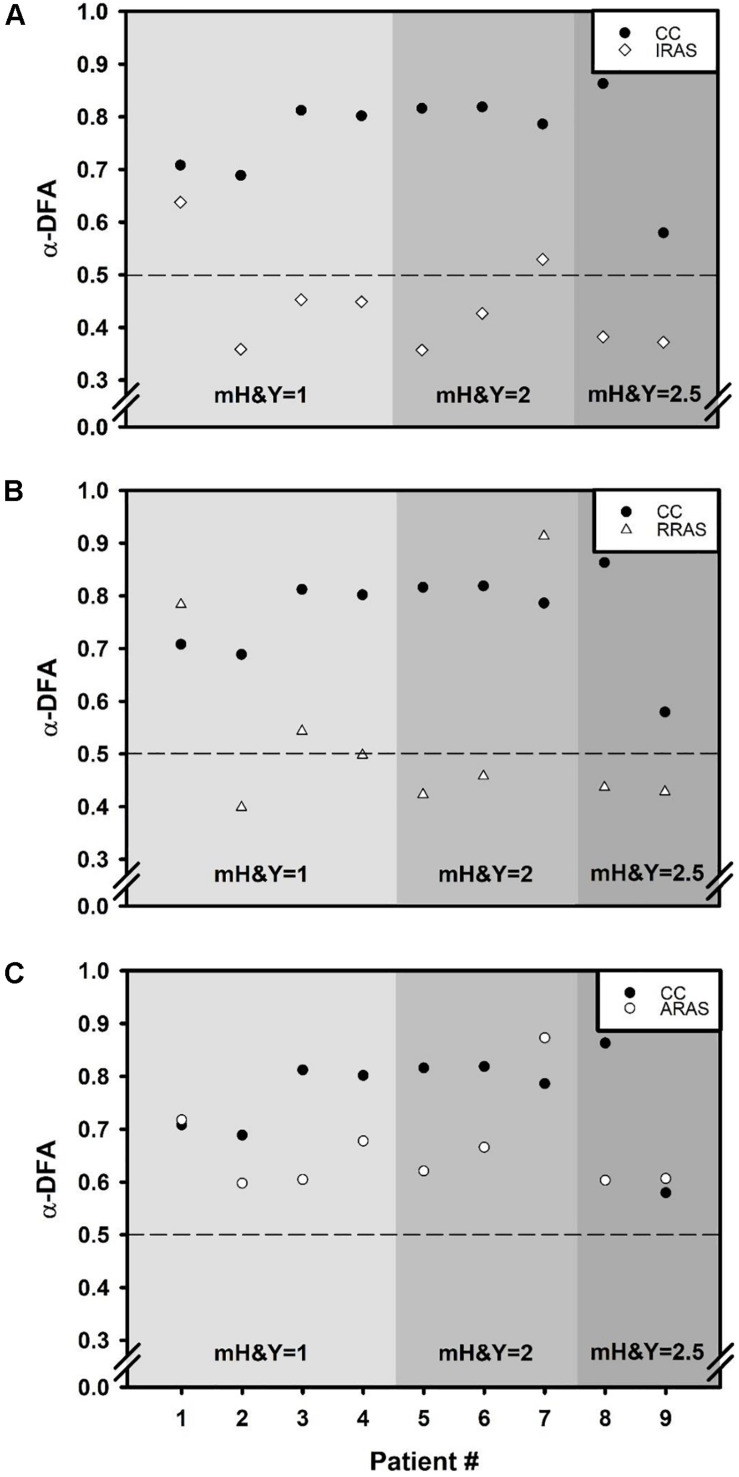
Scatter plots comparing α exponent of each of the nine patients during Control Condition (CC) to α exponents obtained during each walking condition: **(A)** Isochronous Rhythmic Auditory Stimulations condition (IRAS), **(B)** Random Rhythmic Auditory Stimulations condition (RRAS), and **(C)** Autocorrelated Rhythmic Auditory Stimulations condition (ARAS). *X*-axis represents the subject number and the *Y*-axis represents α exponent value during each condition and calculated using the evenly spaced averaged version of the Detrended Fluctuation Analysis (α-DFA). The black dashed line placed at 0.5 on the *Y*-axis delimit the area above which there are LRA. Each graph is divided into three parts representing a stage of the modified Hoehn and Yahr scale (mH&Y) from smallest (left) to largest (right).

Concerning temporal organization of stride duration variability, α-DFA during ARAS was higher than during RRAS and IRAS and was the highest during CC. Indeed, a significant difference was found [*F*(3, 8): 21.487; *p* < 0.001] (Table 2 and Figure 1). LRA were present for all patients during CC (Figure 2). The mean α-DFA was the highest during this condition (0.76 ± 0.09) and within normative data of healthy population according to Z-scores (Table 2 and Figures 1, 2). Also, Cohen’s d was always >1 between CC and the three other RAS conditions, indicating large effect sizes (Figure 1).

During ARAS, LRA were present for all patients (Figure 2) and mean α-DFA (0.66 ± 0.09) was lower than α-DFA during CC but remained above −1.96 Z-scores, indicating LRA within the normative data (Table 2 and Figures 1, 2). Furthermore, α-DFA during ARAS was significantly higher than during IRAS and RRAS.

During IRAS, LRA were lowered (α-DFA = 0.44 ± 0.09) compared to CC and Z-scores were below −1.96 which means below normative data (Table 2 and Figure 1). During this condition, α-DFA was <0.5 for seven patients, meaning anti-persistence. Similarly, α-DFA was close to 0.5 for the last two patients suggesting that stride duration variability was getting closer to complete randomness during IRAS (Figure 2).

During RRAS, α-DFA (0.54 ± 0.18) was significantly lower than during CC, close to 0.5 and not significantly higher than during IRAS for α-DFA (Table 2 and Figures 1, 2). Z-scores were very close to −1.96 Z-scores during this condition suggesting that α-DFA during RRAS was almost out of the normative data (Table 2).

## Discussion

This study investigated the extent to which auditory stimuli with different temporal organizations could influence PD gait. First, this study did not show difference concerning spatiotemporal gait parameters and magnitude of stride duration variability between all conditions. On the contrary, this study highlighted that the three RAS influence the temporal organization of stride duration variability differently. Indeed, LRA were markedly modified during IRAS and RRAS, whereas α-DFA was maintained within normative data and less modified during ARAS.

The spatiotemporal gait parameters were similar between all conditions. These results are reassuring since these parameters have not been degraded during the RAS conditions, whatever the rhythm used. In other studies, PD patients presenting with spatiotemporal gait disorders have improved these parameters using isochronous RAS over several weeks (Arias and Cudeiro, 2008; Dalla Bella et al., 2015). Likewise, improvements were obtained when the RAS had a frequency 10% higher than patients’ spontaneous gait cadence (Dalla Bella et al., 2015).

The analysis of stride duration variability using linear mathematical methods (mean, CV) revealed that the magnitude of the fluctuations was not influenced by the conditions (Table 2). These results are in agreement with those of Uchitomi et al. (2013) but contrary to those of Dotov et al. (2017) and Marmelat et al. (2020) who had shown an increase in the CV.

On contrary, all RAS influenced PD patients’ temporal organization of stride duration variability differently highlighting further the importance of supraspinal centers in the regulation of gait variability given the influence of these interacting external stimulations. LRA were present in all patients during CC with normal α-DFA according to the normative data of healthy population (Warlop et al., 2017; Ravi et al., 2020). This study showed that the use of IRAS led to anti-persistence among seven out of the nine patients. For the others, during IRAS, α-DFA was close to 0.5 indicating a temporal organization close to randomness. As stated in the introduction, it has been shown LRA were positively correlated with balance status (BESTest and ABC scale) (Warlop et al., 2016). In this hypothesis, PD patients’ postural stability could be impaired when α-DFA is lowered. As a corollary, the use of an isochronous metronome would potentially induce greater postural instability for these patients (Hausdorff, 2007). This should be confirmed with longitudinal clinical studies. Unlike during IRAS and RRAS, LRA were present for all patients during ARAS. Although a significant decrease in α-DFA could be demonstrated during this condition compared to CC, the results remained within the normative data oh healthy population (Warlop et al., 2017; Ravi et al., 2020).

Recently, it has been suggested that the presence of LRA in biological systems would represent its healthy status marked by abilities to flexibly adapt to the daily stresses imposed on the body (Goldberger et al., 2002; Stergiou et al., 2006; Hausdorff, 2007). While the metronome is widely used in PD patients’ gait rehabilitation, this study confirmed that it could lead to less persistence in the temporal organization of gait, whatever the rhythm used. According to Stergiou et al. (2006), among other biological signals, healthy gait would present with an “optimal movement variability.” Deviation from this optimal level in either the direction of randomness or over-regularity would represent a loss of adaptative capabilities of the locomotor system (Stergiou et al., 2006; Stergiou and Decker, 2011). The next line of reasoning will follow this theoretical model. Each RAS imposed a rhythm on the patients, a limiting constraint that reduced degrees of freedom during gait. Indeed, patients were asked to synchronize steps with the RAS and had to readjust the timing of each step in relation to the next in accordance with the imposed rhythms. This could therefore explain why α-DFA during each condition is close to that of the different RAS. In this context, the absence of variation of the isochronous metronome would be contrary to the natural fluctuations present in healthy subjects’ gait and compels the patient to synchronize to stereotyped and less complex RAS (Hausdorff, 2007; Kaipust et al., 2013). In the same way, the use of a random metronome would make the temporal organization of gait noisier and more unstable because of a complete lack of structure in the RAS. Both situations are marked by an absence or decrease in persistence. Whether it is an excess of order or complete disorder, this could induce a loss of adaptability in patients’ gait (Stergiou et al., 2006). Then, the compromise between excessive order and disorder would be the use of an autocorrelated metronome. Autocorrelated RAS would allow PD patients to have a necessary structure during walking while giving them a certain freedom in carrying out gait, a repertoire of adaptative motor behaviors for the same situation. This is illustrated by an α-DFA within the normative data of healthy subjects during ARAS and therefore closer to 1. According to this theoretical framework (Peng et al., 1995; Harrison and Stergiou, 2015; Ravi et al., 2020), α-DFA close to 1 (i.e., 1/f noise) would be considered as an optimal state of variability and a sign of a strong coordination between the sub-elements composing the system generating and organizing gait. Therefore, getting closer to 1 could be a rehabilitation goal for these patients. Previous studies supported this assumption since it would be possible to discriminate elderly fallers from non-fallers using LRA computation (Hausdorff, 2007) and as mentioned above, correlations were found between a low α-DFA and poor balance test scores in PD patients (Ota et al., 2014; Warlop et al., 2016). Further longitudinal studies should be conducted to confirm this hypothesis.

Several authors also studied the effects of different RAS on gait variability among healthy young (Kaipust et al., 2013; Marmelat et al., 2014) and old (Kaipust et al., 2013) subjects’ and on PD patients’ LRA (Dotov et al., 2017; Marmelat et al., 2020). On one hand, their results clearly showed that the use of an isochronous metronome lead to less persistence in gait (Kaipust et al., 2013; Marmelat et al., 2014; Dotov et al., 2017). On the other hand, the use of autocorrelated RAS allowed either to maintain α exponent at the level of non-cued gait, or even to have a more persistent stride-to-stride variability. However, these results cannot be compared perfectly with those of the present study since some of these studies (Kaipust et al., 2013; Marmelat et al., 2014) only included healthy subjects and since the acquisition times used were all short to compute LRA in a robust manner (5–6 min).

Dotov et al. (2017) and Marmelat et al. (2020) also tested ARAS on PD patients. As already discussed in the Introduction section, this study differs methodologically from theirs. Indeed, this study differs with the one of Dotov et al. (2017): longer acquisition time, RAS frequency set according to participants’ comfort cadence, known α-DFA used to create ARAS and longer track with less steering. Lastly, Dotov et al. (2017) did not asked patients to synchronize their step to the beats while this was the case for this present study. On the other hand, despite these differences, our results were similar. Unlike our results and those of Dotov et al. (2017), Marmelat et al. (2020) found significantly higher α-DFA values during ARAS (the 1:1 step-to-beat ratio version) compared to their CC. In their study, music was used to deliver ARAS when a simple beat was used in this study. As notified in the Introduction, music is composed of multiple “layers” giving music multiple frequency ranges making it a more complex auditory cue. This greater complexity could offer more degrees of freedom to patients compared to an usual metronome that could constitute a more rigid framework, even with an autocorrelated rhythm organization (Cavanaugh et al., 2017). This could partly explain why Marmelat et al. (2020) noticed an increase in the α-DFA during their ARAS. Also, music evokes emotions (Zatorre et al., 2007), improves motivation (Terry et al., 2012) and it is not currently possible to rule out potential effects of these features on the LRA. The second difference lies in the α-DFA used to produce the ARAS. Indeed, Marmelat et al. (2020) used an α-DFA ∼ 1 which is believed to be an optimal state of variability (Peng et al., 1995; Harrison and Stergiou, 2015; Ravi et al., 2020). The present study opted for an α-DFA = 0.78 representing the natural temporal organization of healthy gait as seen in previous studies (Warlop et al., 2017, 2018) and confirmed by the meta-analysis of Ravi et al. (2020). Based on the results of this study, that of Dotov et al. (2017) and Marmelat et al. (2020), the question that remains is which α-DFA to choose to get the optimal temporal organization of the autocorrelated RAS to be used with PD patients. This should be answered with future transversal and longitudinal studies evaluating the long-term effects of RAS using different α exponents as references.

This pilot study included nine mildly impaired patients with α-DFA within normative data of healthy patients (Warlop et al., 2017; Ravi et al., 2020). It would then be interesting to analyze the influence of the different RAS on a greater number of PD patients and at more advanced stages of the disease. Also, the long-term effect of RAS should be analyzed following a training program to determine whether short-term results are maintained or changed over the long term. Third, no analysis of synchronization between steps and RAS has been performed. This should be done in future similar studies. Lastly, even though the PD patients served as their own control group with CC, no control group composed with healthy subjects was included in this study. One of the prospects for the future is the use of new technologies, such as the smartphone, to produce RAS. These technologies would be able to assess patient’s gait continuously and to deliver ARAS structured with α-DFA adapted in real-time to the patient’s needs and situations as suggested by Hove et al. (2012) and Hristovski and Balagué (2012). Such a system should be tested with PD patients in future studies.

In conclusion, the temporal organization of the RAS has a marked impact on temporal organization of stride duration variability among PD patients. IRAS and RRAS lead to less persistence, whereas ARAS allowed to maintain gait variability closer to baseline. Given the results of this study and those of previous ones, the use of an autocorrelated metronome could therefore be an alternative when proposing auditory cueing to patients. However, future transversal and longitudinal studies must be conducted in order to determine the optimal α exponent used to produce autocorrelated RAS and to investigate the clinical utility of this type of metronome in comparison with each other RAS.

## Data Availability Statement

The raw data supporting the conclusions of this article will be made available by the authors, without undue reservation, to any qualified researcher.

## Ethics Statement

The studies involving human participants were reviewed and approved by the Comité d’Ethique Hospitalo-facultaire of Cliniques universitaires Saint-Luc. The patients/participants provided their written informed consent to participate in this study.

## Author Contributions

AL handled data analysis, interpretation of the results, and wrote the manuscript. TW handled the creation of study protocol, managed patient recruitment, participated in the interpretation of the results, and revised the manuscript. CC helped with patient recruitment, data collection, data analysis, and interpretation of the results. BC provided methodological inputs to the study and participated in the interpretation of the results. GS participated in the interpretation of the results and revised the manuscript. CD provided methodological and statistical inputs to the study, helped to the application of mathematical methods, participated in the interpretation of the results, and revised the manuscript. TL was the chief investigator, provided methodological inputs to the study, participated in the interpretation of the results, greatly assisted in writing the manuscript, and revised the final manuscript. All authors approved the final manuscript.

## Conflict of Interest

The authors declare that the research was conducted in the absence of any commercial or financial relationships that could be construed as a potential conflict of interest.
